# Natural selection increases female fitness by reversing the exaggeration of a male sexually selected trait

**DOI:** 10.1038/s41467-021-23804-7

**Published:** 2021-06-08

**Authors:** Kensuke Okada, Masako Katsuki, Manmohan D. Sharma, Katsuya Kiyose, Tomokazu Seko, Yasukazu Okada, Alastair J. Wilson, David J. Hosken

**Affiliations:** 1 Graduate School of Environmental and Life Science, Okayama City, Okayama Japan; 2 Department of Agricultural and Environmental Biology, The University of Tokyo, Bunkyo-ku, Tokyo Japan; 3 Centre for Ecology & Conservation, University of Exeter, Penryn, UK; 4 National Agriculture and Food Research Organization, Central Region Agricultural Research Center, Tsukuba, Ibaraki Japan; 5 Department of Biological Sciences, School of Science, Tokyo Metropolitan University, Hachiohji, Tokyo Japan

**Keywords:** Experimental evolution, Sexual selection

## Abstract

Theory shows how sexual selection can exaggerate male traits beyond naturally selected optima and also how natural selection can ultimately halt trait elaboration. Empirical evidence supports this theory, but to our knowledge, there have been no experimental evolution studies directly testing this logic, and little examination of possible associated effects on female fitness. Here we use experimental evolution of replicate populations of broad-horned flour beetles to test for effects of sex-specific predation on an exaggerated sexually selected male trait (the mandibles), while also testing for effects on female lifetime reproductive success. We find that populations subjected to male-specific predation evolve smaller sexually selected mandibles and this indirectly increases female fitness, seemingly through intersexual genetic correlations we document. Predation solely on females has no effects. Our findings support fundamental theory, but also reveal unforseen outcomes—the indirect effect on females—when natural selection targets sex-limited sexually selected characters.

## Abstract (web summary)

Natural and sexual selection can be in opposition favouring different trait sizes, but disentangling these processes empirically is difficult. Here Okada et al. show that predation on males shifts the balance of selection in experimentally evolving beetle populations, disfavoring a sexually-selected male trait but increasing female fitness.

## Introduction

Sexual selection typically acts more strongly on males and is responsible for the evolution of a vast array of exaggerated characters that enhance male sexual fitness components^1–4^. Lande’s^5^ and Kirkpatrick’s^6^ models of sexual selection via the Fisher^7^ process—the null models of intersexual selection^8^—clearly shows how this can occur. They also demonstrate how natural selection can oppose sexual selection as trait values move beyond their naturally selected optima (reviewed in ref. ^9^). While theory is clear on the joint effects of natural and sexual selection on sexual trait evolution, explicit experimental tests of theoretical predictions are required to fully understand sexual trait evolution^10^.

One potentially important source of natural selection that could affect the evolution of sexually selected traits is predation^1^, and many studies have shown predation can seemingly oppose the exaggeration of male sexual characters. For example, sexual signals are conspicuous to potential mates but may also attract predators and parasitoid^11^. This is particularly well documented in orthopterans and frogs^12–18^, and this form of natural selection is probably responsible for the loss of cricket sexual signals on two Hawaiian islands^19,20^. More generally, predation appears to reverse the evolution of extreme sexually selected phenotypes (reviewed in the ref. ^21^) and males frequently reduce their sexual signaling in response to predation risk, which can result in decreased mating success when risk is high^22–25^. Nonetheless, while there is ample evidence that predation selects against sexual trait enhancement, there is limited direct experimental verification that this selection causes evolutionary responses in these traits (but see e.g. ^26,27^).

Female reproductive success can also be impacted by predation^28,29^. For example, egg-carrying females can be slower^30,31^ and suffer higher predation rates^29,32^. Resultant anti-predator behaviors may reduce foraging efficiency and reproductive activity, and thus be costly to females (reviewed in the ref. ^22^). Costs can accrue via delayed development, slower growth or postponed reproduction^33–36^. Nonetheless, while there is ample evidence that predation selects on both females and males, the joint action of selection on the sexes is frequently investigated independently. Unfortunately, without exploring how predation affects both sexes, we are unlikely to fully understand how predation affects sexual trait evolution^10^. This is especially true when intersexual genetic correlations link sexually selected male characters with female fitness, because selection on one sex can affect the other through these correlations^37,38^. And again, controlled experimental tests for evolutionary responses to predation are usually not undertaken.

Here, we use the broad-horned flour beetle *Gnatocerus cornutus* to directly test for effects of predation on the evolution of a male sexually selected character (their mandibles) and female lifetime reproductive success (LRS). The enormously enlarged male mandibles are used in male–male fights, and males with larger mandibles have higher fighting and mating success^39–42^. Females lack this exaggerated character completely^40,43^. Previous work has shown that males with larger mandibles sire daughters with lower fecundity, and that directly selecting for increased (or decreased) mandible size results in decreased (or increased) female fitness (LRS)^40,42^. This apparently occurs because of the beetle’s genetic architecture, which means evolving larger mandibles results in the correlated evolution of masculinized females (even though females never develop mandibles). Basically, the enlarged male mandible requires a masculinized head and prothorax to function optimally and this means males with larger mandibles have smaller abdomens^40,44^. Thus although females never develop mandibles, selecting on male mandibles ultimately affects female abdomen size, which likely determines the number of eggs a female carries^40,45^.

These previous *G. cornutus* studies clearly document intralocus sexual conflict over beetle morphology^40,42^ and point to a negative intersexual correlation between (male) mandible size and female fitness, although this link has not been directly established and requires confirmation (c.f.^46^). With this in mind we investigate the beetle’s intersexual genetic architecture for key focal traits using a standard pedigree-based mixed model approach (commonly referred to as an animal model)^47^. We also establish replicate (3/treatment) experimental evolution populations subjected to either male or female predation, along with control populations (*n* = 3, for nine total populations) to investigate how predation affects an exaggerate male trait and female fitness. The assassin bug *Amphibolus venator*, which preys on various stored-product insect pests including flour beetles^48^, is the model predator. After eight generations of experimental evolution, we measure female fitness (LRS) and a range of morphological characters, including mandible size. Morphology was also measured during experimental evolution. We find strong effects of male-specific predation on morphology and female fitness, while predation on females alone had no effects. We additionally test for associations between male mandible size and predation likelihood and find larger mandibles result in greater likelihood of predation, and also show that evolving under predation made males less effective fighters.

## Results

### Genetic architecture

The genetic parameters estimated from the animal model analyses confirmed what previous experimental evolution studies had only inferred^39,40^. Likelihood ratio comparisons of univariate models confirmed the presence of additive genetic variance in all four traits (Supplementary Tables 1–2) as expected. Note that the decision to treat the two mass measures (body/abdomen) as the same trait across the sexes was justified by an absence of significant genotype-by-sex (GxS) interactions in univariate models—a GxS implies sex limited genetic variance is present and can manifest as cross-sex genetic correlations (*r*_Gmf_) <+1 and/or differences in sex specific additive genetic variances. Here *r*_Gmf_ was approximately +1 for both traits, but we do note there is a qualitative pattern of higher additive variance for body mass in females (see Supplementary Tables 1 and 2). Multivariate models further confirmed the presence of additive genetic variance (LRT comparison of null model to one with a diagonal genetic matrix; *χ*^2^_4_ = 74.96, *P* < 0.0001), as well as significant among-trait genetic correlation structure (LRT comparison of a model with a diagonal genetic matrix with all genetic correlations are fixed to zero to the full model in which all genetic correlations are estimated; *χ*^2^_6_ = 39.02, *P* < 0.0001). Parameter estimates from the full multivariate model show substantial genetic variation in all traits measured and reasonably high trait heritability (Table 1). All traits were positively genetically correlated except male mandible size and female fitness (lifetime reproductive success) and male mandible size and abdominal mass, which were both strongly negatively correlation. Individual genetic correlations were nominally significant at *α* = 0.05 (based on |*r*_G_ |> 1.96SE) except those between body and abdomen mass and between body mass and female LRS. The lack of correlation between body and abdomen mass is consistent with previous findings^40^.

**Table 1:** Estimates of the genetic variance-covariance structure among traits in the breeding design.

Traits	Body mass	Abdominal mass	Male mandible size	Female LRS
Body mass	**0.346** (0.085)	0.296 (0.164)	**0.574** (0.162)	0.422 (0.242)
Abdominal mass		**0.513** (0.096)	**−0.415** (0.171)	**0.596** (0.199)
Male mandible size			**0.380** (0.111)	**−0.598** (0.250)
Female LRS				**0.214** (0.106)

Estimates are shown standardized to narrow sense heritability (*h*^2^; bolded diagonal) and genetic correlations (*r*_G_; above diagonal). Values were estimated using a four-trait animal model with body and abdomen mass treated as the same trait in both sexes (but with sex as a fixed effect). Standard errors are denoted in parentheses and bold font denotes estimates that are nominally significant at *P* < 0.05 assuming approximate 95% CI are provided by the estimate ±1.96 SE.

### Predation and evolution

**Fig. 1:**
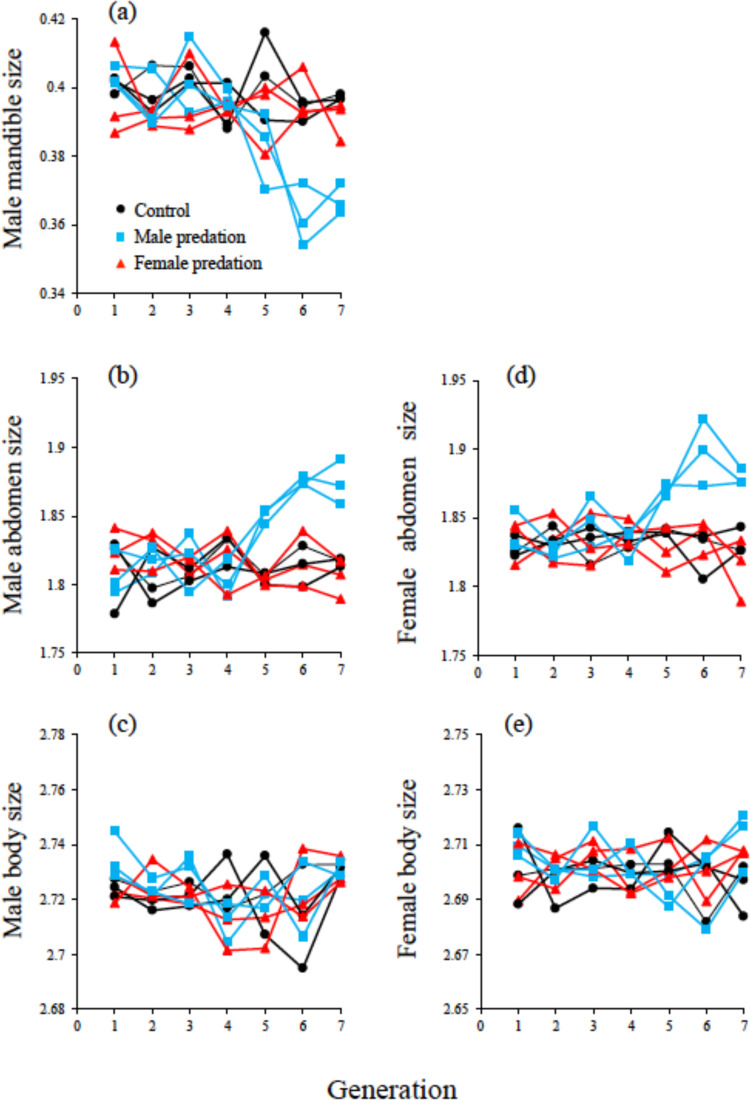
Responses to sex-specific selection through predation over seven generations. Shown are male mandible size (mm) (**a**), male abdomen size (mg) (**b**), male body size (mg) (**c**), female abdomen size (mg) (**d**) and female body size (mg) (**e**) (population means). Black circles are the control populations that were not subjected to selection by predation. Blue squares and red triangles, are the populations with male and female exposure to predators, respectively. Note we did not measure female fitness (lifetime reproductive success: LRS) at every generation as it was not logistically possible. Population means (estimated by measuring 50 male and females per generation), for each population/treatment are shown because only populations evolve and thus population is the biologically relevant unit of replication in an experimental evolution study. Source data are provided as a Source Data file.

**Fig. 2:**
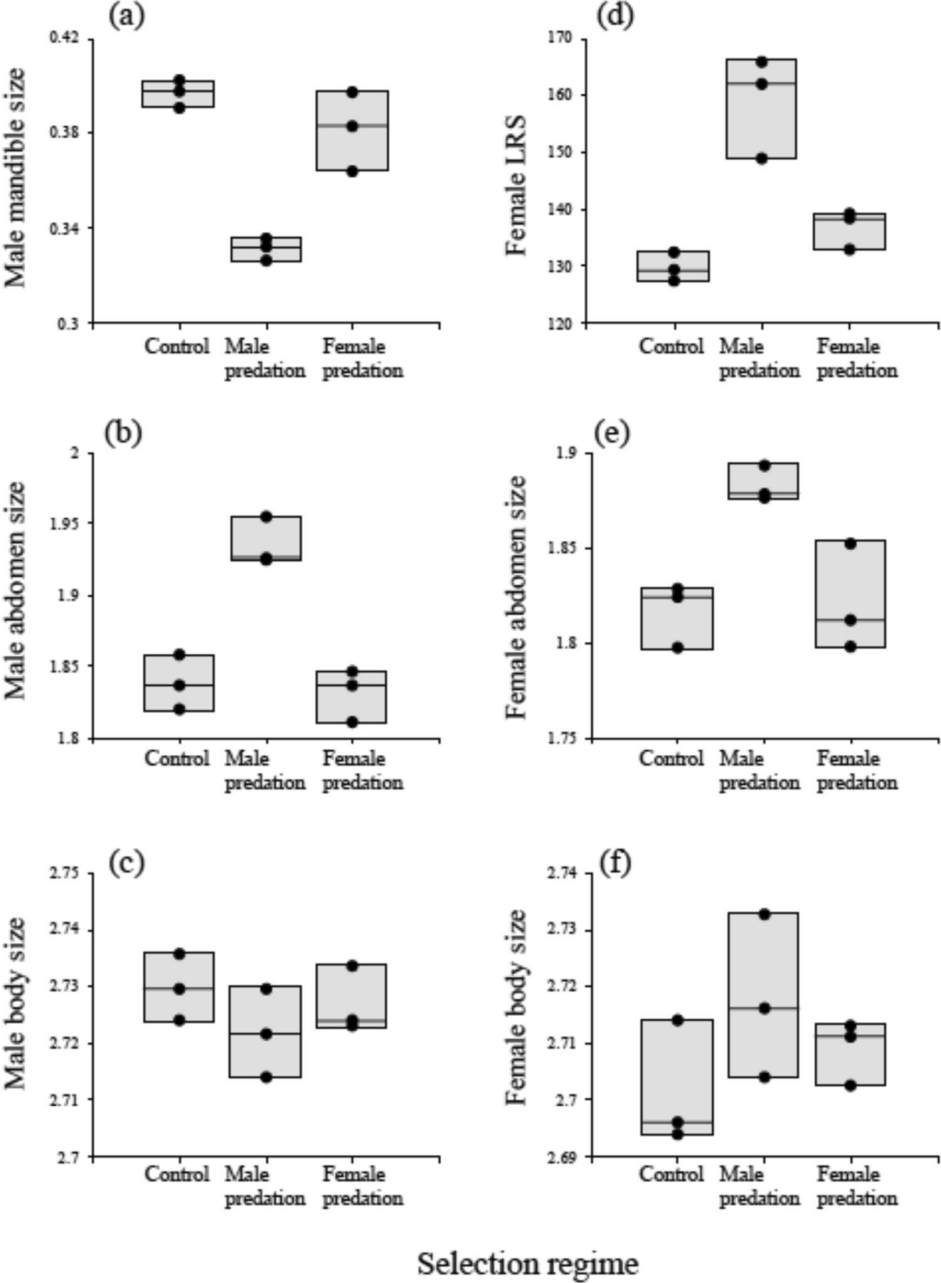
Trait values after eight generations of experimental evolution. Replicate populations (3/treatment) were exposed to either male-only predation (middle columns), female-only predation (right-hand columns) or no predation (controls: left-hand columns), and effects on a range of traits was assessed. Traits were: Male mandible size (mm) (**a**), Male abdomen size (mg) (**b**), Male body size (mg) (**c**), Female lifetime reproductive success (LRS: offspring number) (**d**), Female abdomen size (mg) (**e**), and Female body size (mg) (**f**) (shown are upper and lower quartile (the box) with medians (lines) and each dot represents the mean of one replicate population). Only populations evolve and thus population is the biologically relevant unit of replication in an experimental evolution study. Mean population values were estimated by measuring 20 individual males and female beetles/population. Source data are provided as a Source Data file.

The experimental treatments we imposed on the replicated beetle populations generated treatment specific evolutionary responses in some traits but not others, and responses were not linear (Fig. 1). When we compared trait values at the end of the experimental evolution period, we found that male predation significantly reduced male mandible size (Fig. 2a. Overall treatment effect: *F*_2,6_ = 31.07; *P* < 0.001: post-hoc *t*-tests [with sequential Bonferroni adjustment] revealed that when males were exposed to predators they evolved the smallest mandibles (all *P* < 0.01), while the control and female treatments did not differ in mandible size (*P* = 0.36)). Similarly, male predation resulted in increased male abdomen size (Overall treatment effect: *F*_2,6_ = 31.04; *P* < 0.001): post-hoc *t*-test revealed that males exposed to predators evolved the largest abdomens (all *P* < 0.01) while control and female predator-exposure treatments did not differ (*P* = 1.0) (Fig. 2b). Total male body size was unaffected by our treatments (Fig. 2c: *F*_2,6_ = 1.17; *P* = 0.373). Finally, evolving under predation makes males less effective fighters (*F*_2,6_ = 24.364; *P* = 0.0013) (Supplementary Fig. 1). Post-hoc *t*-tests showed that this was because in the male predation treatment, fighting success was lower (all *P* < 0.01), while the other treatments did not differ (control = female predation: *P* = 0.190).

Predation also affected female LRS (Fig. 2d: *F*_2,6_ = 21.29; *P* = 0.002) (note females mated with “non-evolved” tester males). Post-hoc *t*-tests showed that this was because in the male predation treatment female fitness was higher (all *P* < 0.01), while the other treatments did not differ (control = female predation: *P* = 0.54). Thus exposing males to predators resulted in the evolution of smaller male mandibles and higher female fitness. As with males, female abdomen size also increased under male predation (Fig. 2e. Overall treatment effect: *F*_2,6_ = 10.75; *P* = 0.010): post-hoc *t-*test revealed the male predator exposure group evolved the largest female abdomens (all *P* ≤ 0.01: control = female predation *P* = 1.0)). Finally, female body size was unaffected by our experimental regimes (Fig. 2f. *F*_2,6_ = 1.65; *P* = 0.269).

### Mandible size and predation likelihood

**Fig. 3:**
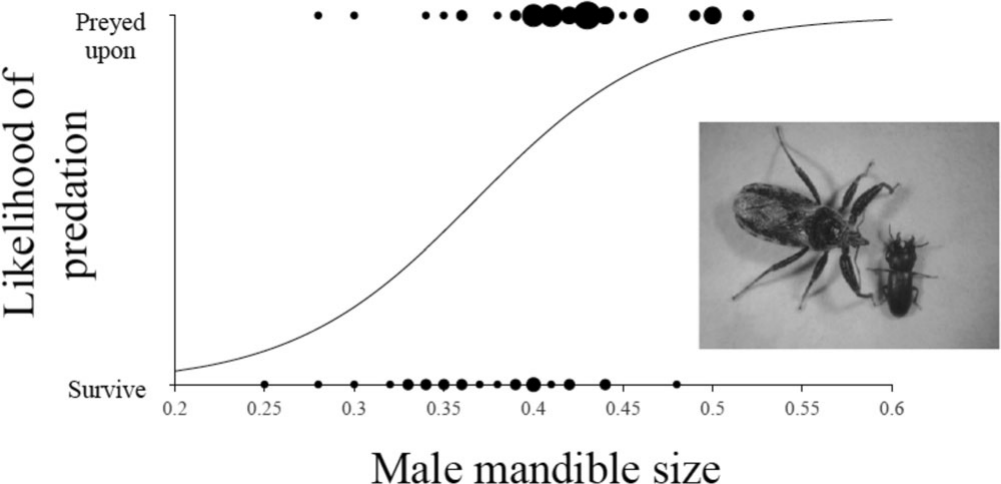
The association between *G. cornutus* mandible size (mm) and likelihood of predation by A. *venator*. The graph shows that as mandible size increases so does the likelihood of predation. The curve is the fitted frequency from the GLM with dot size representing numbers (1–6) of individual *G. cornutus* (i.e., larger dots = more beetles). Inset shows an *A. venator* about to attack a male *G. cornutus*. Note the size difference. Source data are provided as a Source Data file.

In the direct assessment of associations between mandible size and predation probability, 45 of 70 male *G. cornutus* were preyed by female *A. venator* within thirty minutes. The likelihood of being attacked by the predator was positively associated with mandible size (Fig. 3; Intercept (±se) = −7.18 ± 2.34; *χ*^2^ = 12.45; *P* < 0.001. Coefficient (±se) = 19.52 ± 5.89; *χ*^2^ = 15.09; *P* < 0.001). Possible reasons for this are discussed below.

### Main results

**Fig. 4:**
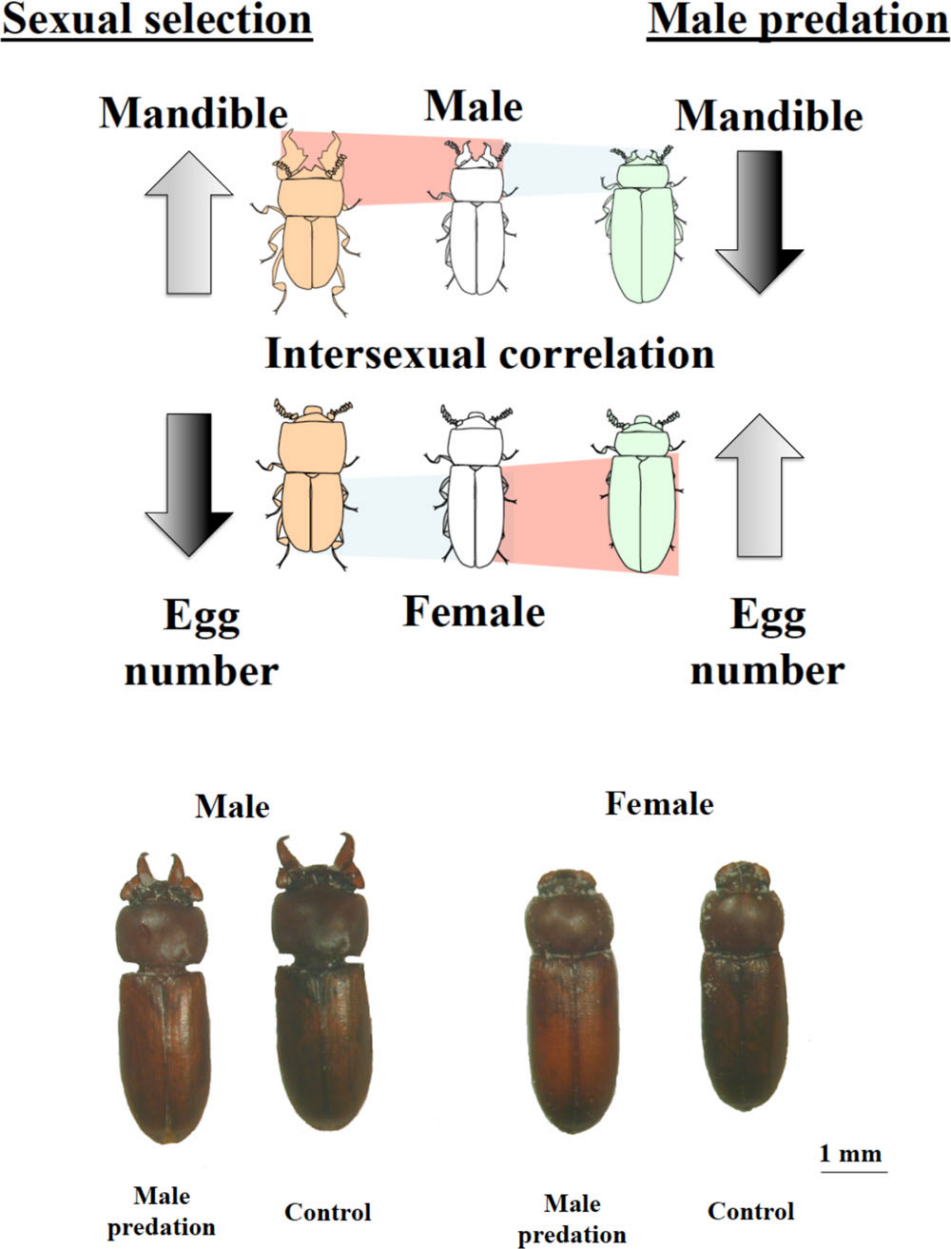
The phenotypes after experimental evolution. The upper panel is a diagrammatic representation of the male and female phenotypes resulting from male-limited predation in comparison to those resulting from sexual selection on males. Sexual selection (left images) results in enlarged male mandibles, which require a masculinized head and prothorax to operate effectively. This fore-body masculinization results in a smaller male abdomen and because of intersexual correlations for abdomen size, a smaller female abdomen and capacity for fewer eggs, even though females never develop mandibles. Male-limited predation selects against the masculinized phenotype, ultimately resulting in larger male and female abdomens, and hence more eggs and higher fitness females (images on the right). The lower plate shows beetles from the male predation and control treatments and reveals the impact of evolving with male predation described above—both males and female evolve more feminized phenotypes (smaller mandibles for males and larger abdomen for both) compared to the controls (NB controls and female-predation have similar phenotypes (Figs. 1 and 2) hence we show only controls for clarity). Images were created by us.

To summarize the main findings: male-specific predation results in the evolution of an altered, more feminized male phenotype that includes reductions in the size of a male-limited sexually selected trait. Additionally, because of the beetle’s genetic architecture, the “new” demasculinized male phenotype is transmitted through to the female phenotype and results in higher fitness females (Fig. 4).

## Discussion

Predation is frequently invoked as an evolutionary brake on the exaggeration of sexually selected traits and there are many studies consistent with this logic, usually documenting selection against larger characters or assessing macro-evolutionary patterns consistent with it^26,27^. Here, we employ experimental evolution and directly demonstrate that male-specific predation not only reversed the exaggeration of a sexually selected trait, but additionally, this reversal results in higher female fitness. This boost to female fitness occurs because predators select against larger mandibles, and this results in a less masculinized beetle phenotype (including a larger abdomen), and because of shared genetic architecture across the sexes, females also become more feminized and produce more offspring. We discuss these findings in turn.

The likelihood of predation increases with male mandible size and when populations were forced to evolve with predation on males, smaller mandibles evolve. Thus increased natural selection via predation reduces the size of a sexually selected trait, which is broadly consistent with theory^5,6^ (reviewed in the ref. ^1^). Previous work with *G. cornutus* suggests why predation has greater impact on males with larger mandibles. Mandible size is negatively phenotypically and genetically associated with locomotor activity^49^, and locomotor activity (running) is a predator escape mechanism in flour beetles^50^. Reduced running and lower escape rates for males with large mandibles would explain the microevolutionary pattern we detect. Interestingly, there is no intersexual correlation for locomotion in this beetle^49^, so there was no expectation that predation on females should impact male running speed and hence morphology. Be that as it may, we clearly show that predation risk increases with mandible size and that this natural selection reverses the evolutionary exaggeration of a sexually selected male trait.

Females from the male-predation populations evolve higher fitness (higher LRS when mating with stock tester males) even though females were not directly exposed to predators. Previously Harano et al.^40^ demonstrated that directly artificially selecting for larger (male) mandibles reduced female fitness. This occurred because selection for increased mandible size resulted in a more masculinized phenotype, and Harano et al.^40^ suggested this masculinization rippled through inferred intersexually-shared genetic architecture to increase the masculinization of the female phenotype, thereby reducing female fitness. The intersexual genetic associations we document here, especially the negative male mandible-female LRS correlation, flesh out this explanation and confirm previous inference^40^. Negative intersexual fitness associations are common^51,52^ because alleles conferring high fitness to one sex frequently lower fitness in the other^53,54^. In addition to any sexually antagonistic selection on body morphology, male beetles with larger mandibles are also more aggressive toward females^55^. Thus exposure to males with larger mandibles potentially reduces female LRS due to (misdirected) male attacks^55^. Therefore, there could be two avenues to increased female fitness when predators select against large male mandibles, a reduction in ontogenetic conflict load (intralocus sexual conflict load^37^), and reduced male-to-female aggression. In any case, predation on males causes an evolutionary reduction in mandible size and this results in higher female fitness. Thus male-biased predation indirectly selects for an increase in female quality, which is an interesting outcome.

The net population level effects of sexual selection and sexual conflict over optimal phenotypes are not clear^51,56^, with for example, evidence that sexual selection can both increase and decrease population extinction rates^57–59^. Additionally, intralocus sexual conflict (as documented in the flour beetle^40^) is thought to constrain population adaptation because sexually antagonistic selection keeps each sex from its fitness optima^51,60^. Our results suggest that predator removal of males with the largest sexual traits reduces intralocus (ontogenetic) sexual conflict costs, enhancing female reproductive performance, which should (all else being equal) increase population productivity. Relaxing other sexual conflict can also increase population fitness^61,62^. It is interesting to note that predators are usually seen as suppressing prey populations^63^, and this can have indirect benefits for ecological competitors of the prey taxon (predation on one species can open up ecological space and lead to higher fitness of the prey’s competitor taxa^64^). As we show, sex-biased predation can have analogous indirect effects intra-specifically (predation on males increases female fitness). However, the indirect impacts we document are unidirectional since female-biased predation does not alter female fitness or male sexual-trait size.

Micro-evolutionary responses to our treatments were not readily apparent until about four or five generations of selection, after which there was clear treatment-specific divergence. Non-linear responses like this are common in selection studies^65,66^ (and see e.g., ^67,68^) and there are many potential genetic and environmental mechanisms that can generate non-linearity. This includes genetic asymmetries (i.e., allele frequencies being unequal), differentially skewed genetic and environmental variation, non-additive genetic variation, and indirect selection^65,66^. We have no way of knowing the mechanistic cause of the patterns we document, but note that responses to directly selecting on mandible size previously revealed similar non-linearity^39^. There have also been many previous studies showing that males with smaller mandibles are less competitive and have lower fitness^39,40^, and that fighting ability is genetically correlated with mandible size^39^. Consistent with these findings, males evolving with male-predation have smaller mandibles and are less adept fighters.

Overall this study provides direct evidence that predator-mediated natural selection can evolutionarily reverse the exaggeration of a sexually selected trait. This finding is consistent with a vast body of fundamental theory^5,6,69^ and empirical evidence from observational and correlational studies^19^ (reviewed in the ref. ^1^). We also reveal interesting outcomes when natural selection targets sex-limited sexually selected characters, since predator removal of a male-imposed conflict load increases female fitness. Thus sex-biased predation within a species can essentially mimic indirect ecological competition effects, as discussed above. Investigating the precise mechanistic detail and population level fitness effects of some of these findings is now required.

## Methods

### *Gnatocerus* stock culture

The *G. cornutus* beetle culture originated from adults collected in Miyazaki City (31° 54′N, 131° 25′E), Japan, and has been maintained in the laboratory of the National Food Research Institute, Japan, for about 50 years on whole meal enriched with yeast as food. The stock is made up of 1500–2000 beetles per generation and maintained in plastic cups (diameter 95 mm, height 50 mm) with a standing density of between 300 and 400 beetles per cup (for a more detailed description of the stock culture, see ref. ^70^). This beetle is a stored product pest, and thus the laboratory conditions very closely mimic what have become their natural conditions. Virgin males and females were removed from the stock population as final instar larvae. Each larva was placed in one well of a 24-well tissue culture plate (Cellstar; Greiner Bio-One, Frickenhausen, Germany) until adult eclosion because pupation in *G. cornutus* is inhibited under high larval density^70^. After eclosion, both sexes were allowed to sexually mature for a period of 14 days prior to their use. We performed all rearing and experiments in a chamber maintained at 25 °C, 60% relative humidity, and with a photoperiod cycle of 14:10 h light/dark. All experiments in this study follow this protocol unless stated otherwise.

### The predator

The assassin bug *Amphibolus venator* is predator of stored-product insect pests and preys on various stored-product insect pests including flour beetles^48,71,72^. These predators are frequently found in stored product facilities, which are the habitat of *G. cornutus*^72^ (and see Fig. 3). The *A. venator* culture originated from adults collected in a storehouse in Urasoe City, Okinawa, Japan, and has been maintained in the laboratory for about 5 years. The stock was initiated and maintained at 200 bugs per generation and housed in plastic containers (230 mm × 150 mm × 80 mm) with a standing density of between 30 and 40 bugs per cup. Each nymph was given an excess of food (seven final instar larvae of *G. cornutus* per week). Each adult female was allowed to mate with a male chosen randomly and to lay eggs in order to maintain the predator stocks. The predatory behavior of *A. venator* follows a stereotypical sequence: they recognize, chase, and capture prey (here *G. cornutus*) using their enlarged forelegs (similar to praying mantis)^48,73^.

### *Gnatocerus* breeding design and estimation of quantitative genetic parameters

Using a full sib/half sib experimental design, males (sires) (*N* = 35) were randomly assigned to three virgin females (dams) (all collected from the stock population). Pairs were housed in a plastic container (17 mm diameter, 20 mm high) containing filter paper (17 mm diameter), and successful copulation was indicated by a stable end-to-end connection between the male and female. After mating, dams were immediately removed and individually placed in a plastic cup (70 mm diameter, 25 mm high) containing excess food (20 g). Each female was housed thus for two months to obtain offspring. All offspring from each female were reared to final instar (approximately 8 weeks). Three sons and three daughters per dam per sire (all pairings produced sufficient young) were haphazardly chosen for measurement of male traits (mandible, body, and abdomen size) and female traits (LRS, body, and abdomen size) at 14 days after eclosion (*N* = 315 per sex) (trait measurement protocols below). Although we were primarily interested in the genetics of male mandible size and female fitness, we included both abdomen size and total body size measures. This is because previous work^40^ found that directly selecting for larger mandibles caused a correlated decrease in abdomen size, but had no effect on total body size. This suggests a trade-off between abdomen and prothorax size and sexual conflict over body shape.

Data from the breeding design were then analysed using pedigree-based animal models fit in ASReml-R^74^. First, to confirm the presence of additive genetic variance in each trait we fit a series of univariate animal models to (male) mandible, body and abdomen size, and to female (LRS). For each trait we compared the model fit to a reduced model with no additive genetic effects using a likelihood ratio test (LRT; adjusted for boundary conditions following^75^). We elected to combine male and female records for both body size and abdomen sizes as additional modeling provided little support for genotype-by-sex interactions (see “Results” section). However, for these traits a fixed effect of sex was included, as well as the (random) additive genetic effect since exploratory analysis showed sexual dimorphism in both traits (body size, males are 0.020 mg (SE 0.005) larger on average, *t* = 3.58, *P* < 0.001; abdomen size, males are 0.019 mg (SE 0.008) smaller on average, *t* = 2.463, *P* = 0.014)).

We then fitted a multivariate animal model to estimate genetic correlation (*r*_G_) structure among the four traits with fixed effects of sex on body size, abdomen size, as well as their heritability (*h*^2^). The residual covariance structure was modeled as an unstructured matrix (but note residual covariance between the sex-limited traits of male mandible size and female LRS is not estimable from the data so was fixed to zero). We also ran reduced multivariate models with i) no genetic effects at all, and ii) a diagonal genetic variance matrix (i.e., genetic variance modeled on all traits but all genetic correlations assumed to equal zero) for comparison to the full model by LRT. This allows statistical inference at the level of the multivariate phenotype. We used estimated standard errors (SE) as a guide to nominal significance of pairwise genetic correlations (assuming approximate 95% CI are given by *r*_G_ ± 1.96SE).

### *Gnatocerus* experimental evolution protocol—sex specific predation

**Fig. 5:**
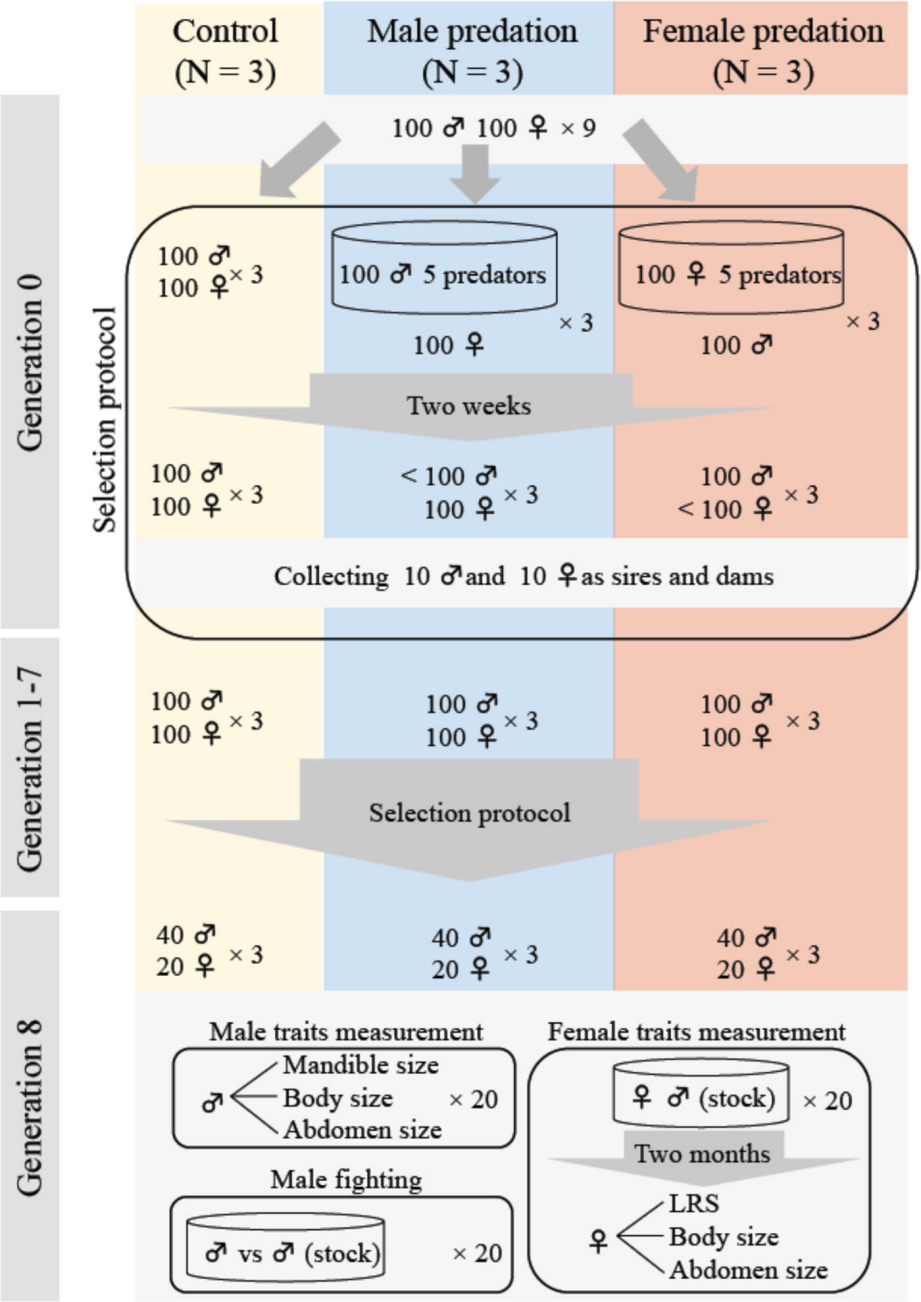
A diagrammatic representation of the experimental evolution protocol. Included is the fighting assay after eight generations of experimental evolution. *N* refers to the number of replicate populations/treatment.

We first collected 900 male and 900 female *G. cornutus* from the stock culture and haphazardly generated nine groups of 100 males and 100 females to establish three male-predation populations, three female-predation populations and three control (no predation) populations (generation 0) (Fig. 5). To simulate predation, 100 males (or females) were housed in a plastic container (150 mm diameter, 50 mm high) containing an excess of beetle food (45 g). Then, five adult female *A. venator* (20–35 day olds) were randomly collected from the predator culture and placed into the container and the males (females) were exposed to them for two weeks. We then selected ten of the males (females) that survived the 2 weeks to act as sires (dams) of the predation treatments—ten opposite sex individuals were also taken/population to act as the non-selected dams (sires) that were not exposed to predation. We note that survival rate during this predation protocol was approximately 20%. To propagate control populations, ten males and ten females were haphazardly selected per population to act as sires and dams. For each population/treatment the ten males and females were placed in a plastic cup (diameter 95 mm, height 50 mm) with 70 g of medium for 2 months, with males able to mate with females and females were allowed to lay eggs, until final instar larvae were obtained. Final instar larvae were collected to obtain the adults for subsequent generations. When the adults reached 14 days old, 100 males and 100 females per population were randomly selected to (potentially) seed the next generation, and in the predation treatments, exposed to predators as above. We then selected surviving animals as above and repeated for eight generations. Additionally, we also collected 50 males and 50 females per population from generation 1 to 7 to assess mandible, abdomen and body size responses to selection. At generation 8, 20 males and 20 females per population were haphazardly collected for measurement of male traits (mandible, body, and abdomen size) and female traits (LRS, body and abdomen size) (*N* = 180 per sex) (trait measurement protocols below).

### Fighting ability

We also conducted male–male fights between evolved males and males from stock populations (Fig. 5). Briefly, after generation eight of experimental evolution, males from the nine populations (control, male predation, and female predation) were collected. We observed 20 contests per population (*n* = 180 in total) in which focal experimental males competed against a male collected from the stock culture using standard methods^39,76^. In brief, the stock males were marked with white spots [Mitsubishi Paint-Marker] on their elytra for identification. Males 14 days old (after final eclosion) were used for the experiments. Before pairing, males were weighed with the electronic balance. To control for the effect of body size on fighting success, males were paired so that the difference in body size between contestants was less than 0.05 mg. Pairs were placed on filter-paper (17 mm diameter) in a plastic container (17 mm diameter, 20 mm high) and allowed to interact (and fight) for 1 h. Previous work has shown that male fights occur in all trials when staged in this manner^76^. Males that pushed opponents and chased them were denoted the winner^76^. Trials were then continuously observed until fight outcomes could be scored.

### Predation and mandible size

To more directly test for possible associations between predation and mandible size, seventy adult female *A. venator* (20–35 days old) were randomly collected from the stock culture and were individually aspirated into in a plastic dish (90 mm diameter, 10 mm high) lined with a filter paper. After 30 min, one male adult *G. cornutus* collected from the stock culture (14 days old: mandible length measured prior to exposure) was added to each dish, and subsequent predation behavior was continuously observed for 30 min.

### Male trait measurement

We measured overall body mass and the posterior body mass (i.e., mesothorax, metathorax, and abdomen) as an abdomen size indicator^40^ (and see comments above. Briefly, each male was frozen at −20 °C immediately after adult emergence. Mass measures were obtained to the nearest 0.01 mg on an electronic balance (Mettler-Toledo AG, Laboratory and Weighing Technologies). The mandible length (±0.01 mm) of each male was measured (±0.01 mm) using a dissecting microscope monitoring system (VM-60; Olympus, Tokyo, Japan). Each specimen was positioned so that its longitudinal and dorsoventral axes were perpendicular to the visual axes of the microscope eyepiece (see ref. ^70^ for landmarks).

### Female trait measurement

To obtain LRS (lifetime reproductive success: our fitness proxy) each female (14 days of post-eclosure) was individually paired with a haphazardly selected male from the stock culture. After copulation, each female was maintained in a plastic cup (70 mm diameter, 25 mm high) containing excess food (20 g) for two months and allowed to lay eggs. This schedule was chosen because most eggs are laid by females within 2 months of mating^77^, and thus this is an accurate index of LRS^42,78^. To measure the LRS of each female, we counted all adults that emerged in the third month after pairing. After the laying period, each female was frozen at −20 °C. Subsequently, the whole and posterior of the body (body size and abdomen size) were weighed with the electronic balance (as above).

### Analysis

Apart from the genetic parameter estimation with an animal model (using ASReml-R as described above), all analyses were conducted using JMP for Windows version 8^79^. For the experimental evolution, we used population as the unit of replication (=9 DF max.) with single fixed factor (with three levels: the experimental treatments) GLMs for each trait to test for effects of experimental evolution, with post-hoc testing for factor-level differences (note we did not have sufficient DF for a multivariate analysis). Results are as reported even after (conservative^80^) sequential Bonferroni correction. Fighting results were compared (as population rates of winning) using a generalized linear model (GLM) (JMP 7)^76^. To directly assess how mandible size affected predation probability we used a GLM with a binomial distribution, a logit-link function, and overdispersion test. Male mandible size was used as the explanatory variable, and predation impact (preyed upon = 1, survive = 0) was the response variable.

### Reporting summary

Further information on research design is available in the Nature Research Reporting Summary linked to this article.

## Supplementary information

Supplementary Information

Description of Additional Supplementary Files

Supplementary Data 1

Reporting Summary

## Data Availability

The data that support the findings of this study are provided in Supplementary Data 1. This includes population mean trait values during and on completion of experimental evolution, fighting data, predation-mandible size data and the pedigree data. Source data are provided with this paper.

## Source data

Source Data
