# Supplementary material for: Natural selection increases female fitness by reversing the exaggeration of a male sexually selected trait
Source: Nat Commun. 2021 Jun 8;12:3420. doi: 10.1038/s41467-021-23804-7 (PMC8187464; doi:10.1038/s41467-021-23804-7)
Supplement: Supplementary file 1 — Supplementary Information [file 41467_2021_23804_MOESM1_ESM.pdf]

Supplementary Information

Natural selection increases female fitness by reversing the exaggeration of a male sexually selected trait

Okada, Katsuki et al.

Supplementary Table 1. Estimated fixed effects and heritabilities from univariate animal models of each trait. Body mass and abdomen mass were treated as single traits rather than sex-specific ones, with sex included as a fixed factor. Statistical inference on fixed effects is by conditional F test while the presence of additive genetic variance was tested by LRT comparison to a reduced model assuming twice the difference in model log-likelihoods is distributed as a 50:50 mix of $X^2{}_0$ and $X^2{}_1$.

	Fixed effects					Random effects		
Trait	Effect	Estimate (SE)	DF	F	P	h^2 (SE)	$X^2{}_{0,1}$	P
Body mass	mean	2.701 (0.005)	1,50.9	318800	<0.001	0.343 (0.085)	33.95	<0.001
	Sex (male)	0.020 (0.005)	1,528.7	15	<0.001			
Abdomen mass	mean	1.843 (0.008)	1,57.2	55940	<0.001	0.508 (0.096)	58.11	<0.001
	Sex (male)	-0.019 (0.007)	1,523.0	8	0.005			
Male mandible length	mean	0.396 (0.004)	1,44.0	10050	<0.001	0.286 (0.121)	8.50	0.002
Female LRS	mean	141.873 (1.714)	1,41.8	6849	<0.001	0.165 (0.108)	3.234	0.036

Suplementary Table 2. Tests for genotype-by-sex interaction in body and abdomen mass. Shown are likelihood ratio test (LRT) comparisons of a simple univariate animal model (including a fixed effect of sex) to once in which genotype-by sex interaction is modelled. Twice the difference in log-likelihoods is assumed to be distributed as X^2 with 2DF. Also shown are estimates of genetic variance (V_A) under the simple model and the sex-specific genetic variances (V_{Af}, V_{Am}) and the cross-sex genetic correlation (r_{Gmf}) under the expanded model allowing genotype-by-sex interaction. Standard errors are shown in parentheses where available. Note that to keep the genetic variance-covariance matrix in allowable parameter space (i.e. positive definite) r_{Gmf} was bound to (effectively) +1 in both expanded models and no SE is estimated as a consequence. For body mass the improvement to model fit is marginally non-significant when the genotype-by-environment interaction is included. To the extent this might reflect real differences in sex-specific genetic architecture the pattern is driven by apparent differences in V_A across the sexes (rather than deviation from r_{Gmf}=1). Consequently, we assuming an absence of genotype-by-sex interaction for this trait, we also note that the estimated heritability presented in the main manuscript remains valid (as an average across the sexes) even if this assumption is incorrect.

	Model comparison		Simple animal model	Expanded model with genotype-by-sex interaction		
Trait	X^2_2	P	V_A (SE)	V_{Af} (SE)	V_{Am} (SE)	r_{Gmf}
Body mass	5.49	0.075	0.0017 (0.0005)	0.0026 (0.0006)	0.0013 (0.0005)	0.9999
Abdomen mass	0.20	0.915	0.0050 (0.0012)	0.0053 (0.0014)	0.0047 (0.0013)	0.9999

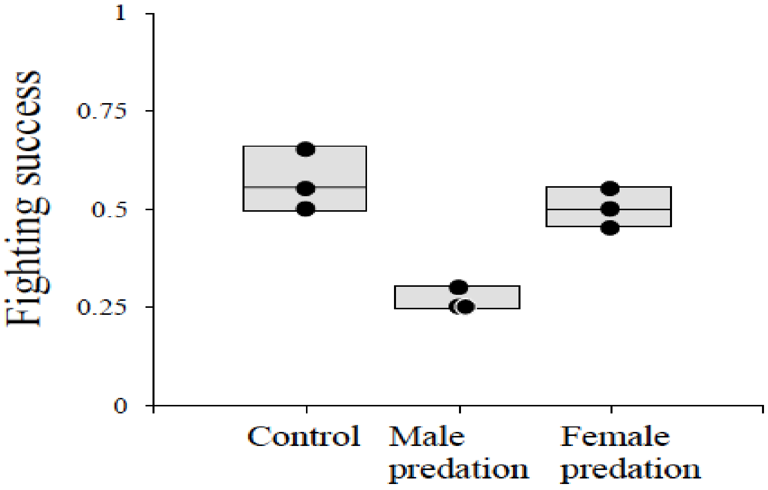

Supplementary Figure 1. The fighting success of males from the three experimental treatments. Males from populations that had evolved with male-only predation were less adept fighters. This is consistent with their smaller mandibles as previous studies have shown that males with larger mandibles are more likely to win fights and that mandible size and fighting ability are genetically correlated[1,2]. Shown are upper and lower quartile (the box) with medians (lines) and each dot represents the mean of one replicate population (3/treatment). NB populations are the biologically meaning replicates in an experimental evolution study. Source data are provided as a Source Data file.

Supplementary References

1. Okada, K. & Miyatake, T. 2009. Genetic correlations between weapons, body shape and fighting behaviour in the horned beetles *Gnatocerus cornutus*. *Anim. Behav*. 77:1057-1065.
2. Okada, K., Okada, Y., Dall, S.R.X. & Hosken, D.J. 2019. Loser-effect duration evolves independently of fighting ability. *Proc. R. Soc. B* 286:20190582.
