# Supplementary material for: Natural selection increases female fitness by reversing the exaggeration of a male sexually selected trait
Source: Nat Commun. 2021 Jun 8;12:3420. doi: 10.1038/s41467-021-23804-7 (PMC8187464; doi:10.1038/s41467-021-23804-7)
Supplement: Supplementary file 2 — Description of Additional Supplementary Files [file 41467_2021_23804_MOESM2_ESM.pdf]

Description of Additional Supplementary Files

File Name: Supplementary Data 1
Description: This includes all the data associated with the paper
