# Supplementary material for: Natural selection increases female fitness by reversing the exaggeration of a male sexually selected trait
Source: Nat Commun. 2021 Jun 8;12:3420. doi: 10.1038/s41467-021-23804-7 (PMC8187464; doi:10.1038/s41467-021-23804-7)
Supplement: Supplementary file 4 — Reporting Summary [file 41467_2021_23804_MOESM4_ESM.pdf]

nature research

Corresponding author(s): David Hosken

Last updated by author(s): 11 May 2021

Reporting Summary

Nature Research wishes to improve the reproducibility of the work that we publish. This form provides structure for consistency and transparency in reporting. For further information on Nature Research policies, see our Editorial Policies and the Editorial Policy Checklist.

Statistics

For all statistical analyses, confirm that the following items are present in the figure legend, table legend, main text, or Methods section.

n/a	Confirmed	
☐	☒	The exact sample size (*n*) for each experimental group/condition, given as a discrete number and unit of measurement
☐	☒	A statement on whether measurements were taken from distinct samples or whether the same sample was measured repeatedly
☐	☒	The statistical test(s) used AND whether they are one- or two-sided<br>*Only common tests should be described solely by name; describe more complex techniques in the Methods section.*
☐	☒	A description of all covariates tested
☐	☒	A description of any assumptions or corrections, such as tests of normality and adjustment for multiple comparisons
☐	☒	A full description of the statistical parameters including central tendency (e.g. means) or other basic estimates (e.g. regression coefficient) AND variation (e.g. standard deviation) or associated estimates of uncertainty (e.g. confidence intervals)
☐	☒	For null hypothesis testing, the test statistic (e.g. *F*, *t*, *r*) with confidence intervals, effect sizes, degrees of freedom and *P* value noted<br>*Give P values as exact values whenever suitable.*
☒	☐	For Bayesian analysis, information on the choice of priors and Markov chain Monte Carlo settings
☒	☐	For hierarchical and complex designs, identification of the appropriate level for tests and full reporting of outcomes
☒	☐	Estimates of effect sizes (e.g. Cohen's *d*, Pearson's *r*), indicating how they were calculated

Our web collection on statistics for biologists contains articles on many of the points above.

Software and code

Policy information about availability of computer code

Data collection	No software were used in data collection.
Data analysis	Provided: ASREML-R Version 4 and JMP8 as reported in the manuscript.

For manuscripts utilizing custom algorithms or software that are central to the research but not yet described in published literature, software must be made available to editors and reviewers. We strongly encourage code deposition in a community repository (e.g. GitHub). See the Nature Research guidelines for submitting code & software for further information.

Data

Policy information about availability of data

All manuscripts must include a data availability statement. This statement should provide the following information, where applicable:

- Accession codes, unique identifiers, or web links for publicly available datasets
- A list of figures that have associated raw data
- A description of any restrictions on data availability

The data that support the findings of this study are provided in Supplementary Data 1. This includes population mean trait values during and on completion of experimental evolution, fighting data, predations-mandible size data and the pedigree data. Source Data are provided with this paper.

Field-specific reporting

Ecological, evolutionary & environmental sciences study design

All studies must disclose on these points even when the disclosure is negative.

Study description	Pedigree analysis and experimental evolution of a beetle.
Research sample	All detail are provided in the MS. In brief, we used a beetle model as it has an obvious sexually selected trait and we can easily replicate what has become its natural environment in the lab (it is a stored product pest). We used one of its natural predators as the the experimental predator. We have worked with this model for more than 10 years.
Sampling strategy	In the experimental evolution, evolving populations are the unit of replication. We replicated population within treatment as much as was physically possible given logistical (workload) constraints. We had 9 experimental populations in total, which exceeds N for many experimental evolution studies (eg Hosken & Ward Ecology Letters 2001). For the pedigree analysis we used a standard genetic design and based on past experience estimated that the 35 sire, 3 dams/sire and 6 offspring per dam would enable us to estimate genetic parameters effectively (which we could: see animal model results). All sampled animals (those to be measured) were chosen haphazardly from those available (ie sons/daughters from the pedigree; males/females from experimental evolution).
Data collection	All details are provided in the MS. Briefly, colleagues in Japan conducted the experimental evolution and collected these data. MD Sharma collected pedigree data in the UK (see author contributions). Beetles were haphazardly collected and traits measured as described (using standard techniques: microbalance for mass and microscope for linear). The pedigree was generated by mating haphazardly selected to sires to dam and then collecting the offspring for measurement - that is we experimentally created the pedigree. Offspring were measured as with other individuals. The team in Japan lead by KO collected evolution data, the genetic data was collected in the UK by MDS.
Timing and spatial scale	The only timing issue was that it was not possible to measure female fitness at every generation of the experimental evolution (as reported in the MS) - it was just too time consuming to care for evolving populations and their treatment manipulations. Therefore this trait was only measured once at the end of the evolution period. Spatially, we split the work across two labs. Pedigree work was done in the UK, experimental evolution in Japan, but the UK stocks were supplied from Japan (ie we were working on the same lab population). Data collection began in 2011 through to final elements conducted in 2021. Animals were originally collected from sites in Japan, 5 or 50 years ago. The G. cornutus beetle culture originated from adults collected in Miyazaki City (31° 54′N, 131° 25′ E), Japan. The A. venator culture originated from adults collected in a storehouse in Urasoe City, Okinawa, Japan, and has been maintained in the laboratory for about 5 years. This information is in the MS.
Data exclusions	No data were excluded.
Reproducibility	Experimental evolution involved replicated populations. If populations did not evolve consistently wrt treatment there would be no effects detected. Pedigree analysis is always population and time specific - it just asks do these offspring look like their parents due to shared genes (or environments). However, the outcomes we detected are consistent with a vast body of published work on these animals (eg previous selection outcomes match the genetic correlations we detected) and trait values (measure blind across labs) were equivalent (control populations vs pedigree data). The experimental evolution treatments had 3 replicates/treatment (N= 9 populations) evolving over 8 generations. Means/replicate were generated by measuring 40-100 animals. Predation was measured on 70 animals. These data are in the MS.
Randomization	Animals were haphazardly allocated to experimental populations - this approach was effective as evidenced by trait values being not statistically different across treatments at Generation 1 of the experiment. See Figure 1 in MS.
Blinding	Not all data collectors were involved in data analysis and due to the nature of the analysis it would not be possible to a priori "know" what outcomes could be. For example, we used Reml of the pedigree to estimate genetic covariances across the sexes on a sample of 630 offspring. It is inconceivable that results could be anticipated during measurement, and in any case there is no expectation of what values should be (no right or wrong answer: the associations are the associations). Similarly, in the experimental evolution, we had 9 populations, and 4 experimenters collected and measured trait values on ca. 100 animals per population/generation. Prior to analyses these values were averaged for each sex (as population not individual is the unit of replication in an evolution study) by population and treatment level and effects were subsequently estimated. Again, it is difficult to imagine how biasing in collection, especially given the small size of the beetles (ie to the naked eye any difference would be impossible to detect), or measurement (individual values of males and females measured independently and then averaged) could affect analytical (GLM) outcomes. Finally, data from each lab were collected blind relative to the other lab. Despite this, trait values were very similar (e.g. control experimental evolution populations vs. pedigree data) across the labs.

Did the study involve field work? ☐ Yes ☒ No

Reporting for specific materials, systems and methods

We require information from authors about some types of materials, experimental systems and methods used in many studies. Here, indicate whether each material, system or method listed is relevant to your study. If you are not sure if a list item applies to your research, read the appropriate section before selecting a response.

Materials & experimental systems

n/a	Involved in the study
☒	☐ Antibodies
☒	☐ Eukaryotic cell lines
☒	☐ Palaeontology and archaeology
☐	☒ Animals and other organisms
☒	☐ Human research participants
☒	☐ Clinical data
☒	☐ Dual use research of concern

Methods

n/a	Involved in the study
☒	☐ ChIP-seq
☒	☐ Flow cytometry
☒	☐ MRI-based neuroimaging

Animals and other organisms

Policy information about studies involving animals; ARRIVE guidelines recommended for reporting animal research

Laboratory animals	Yes all included in the manuscript. The A. venator culture originated from adults collected in a storehouse in Urasoe City, Okinawa, Japan, and has been maintained in the laboratory for about 5 years. The G. cornutus beetle culture originated from adults collected in Miyazaki City (31° 54′N, 131° 25′ E), Japan, and has been maintained in the laboratory of the National Food Research Institute, Japan, for about 50 years.
Wild animals	No wild animals were used.
Field-collected samples	The A. venator culture originated from adults collected in a storehouse in Urasoe City, Okinawa, Japan, and has been maintained in the laboratory for about 5 years. The G. cornutus beetle culture originated from adults collected in Miyazaki City (31° 54′N, 131° 25′ E), Japan, and has been maintained in the laboratory of the National Food Research Institute, Japan, for about 50 years. We did not collect ourselves.
Ethics oversight	Yes all ethical considerations were met according to the University of Exeter and University of Okayama regulations.

Note that full information on the approval of the study protocol must also be provided in the manuscript.
